# Posttransplant Anemia as a Prognostic Factor of Mortality in Kidney-Transplant Recipients

**DOI:** 10.1155/2017/6987240

**Published:** 2017-03-19

**Authors:** Maria Majernikova, Jaroslav Rosenberger, Lucia Prihodova, Miriam Jarcuskova, Robert Roland, Johan W. Groothoff, Jitse P. van Dijk

**Affiliations:** ^1^Fresenius Medical Care-Dialysis Services Slovakia, Kosice, Slovakia; ^2^Graduate School Kosice Institute for Society and Health, Faculty of Medicine, Safarik University, Kosice, Slovakia; ^3^Department of Health Psychology, Faculty of Medicine, Safarik University, Kosice, Slovakia; ^4^Transplantation Department, Faculty of Medicine and University Hospital, Safarik University, Kosice, Slovakia; ^5^St. Lukas Geriatric Centre, Kosice, Slovakia; ^6^Department of Community & Occupational Health, University Medical Centre Groningen, University of Groningen, Groningen, Netherlands

## Abstract

*Background.* Findings on the association between posttransplant anemia (PTA) and mortality in posttransplant patients are scarce. This study explored whether PTA shortly after kidney transplantation (KT) predicts mortality at up to 10 years' follow-up, stratified for chronic kidney disease (CKD) stages.* Methods.* PTA was divided into 3 categories according to the hemoglobin (Hb) value: severe (Hb < 10 g/dl), mild (10.0 g/dl ≤ Hb < 11.9 g/dl), or no PTA (Hb ≥ 12 g/dl). CKD stages were estimated using the CKD-EPI formula and divided into 2 groups: CKD1-2 and CKD3–5. Cox regression, stratified according to CKD, was performed to identify whether different categories of PTA predicted mortality in KT recipients.* Results.* Age, being female, and both mild and severe PTA contributed significantly to the Cox regression model on mortality in CKD1-2. In the Cox regression model for mortality in CKD3–5, age and severe PTA contributed significantly to this model.* Conclusion.* PTA shortly after KT increased the risk of mortality at up to 10 years' follow-up. Even mild PTA is associated with a 6-fold higher risk of mortality and severe PTA with a 10-fold higher risk of mortality in CKD1-2. Clinical evaluation and treatment of anemia might reduce the higher risk of mortality in patients with PTA in early stages of CKD after KT.

## 1. Introduction

The definition and grades of anemia were established decades ago by the World Health Organization (WHO) as being among the important factors influencing health outcomes: decreased hemoglobin concentration predicts morbidity and mortality in the general population [1], and this definition was consequently adopted by nephrologists. According to “The National Kidney Foundation Disease Outcomes Quality Initiative” (NKF/KDOQI), “Kidney Disease Improving Global Outcomes” (KDIGO), and “European Best Practice Guidelines” (EBPG), anemia is defined as a target hemoglobin (Hb) <13.5 g/dl in adult males/postmenopausal females, <12.0 g/dl in premenopausal females, and <5th percentile for children [2–4]; alternatively, the target Hb should generally be <11.0 g/dl [5, 6].

However, in most individuals there is a considerable amount of variation in the Hb-value over time, and the consequences of this variability in Hb-levels have been thoroughly studied in dialysis patients, though not in transplant recipients [7]. Renal anemia after transplantation, or posttransplant anemia (PTA), has a multifactorial etiology including the progress of transplant kidney failure, comorbidity, infections, inflammation, angiotensin-converting enzyme inhibitor/angiotensin receptor blocker (ACEi/ARB), and immunosuppressive treatment [2–6, 8–10]. Thus far, some evidence has been found suggesting that kidney-transplant recipients may have Hb-level lower than what can be expected based on the level of their kidney function [2].

Guidelines from NKF/KDOQI, KDIGO, and EBPG recommend treating anemia of renal origin in order to reduce both morbidity and mortality [2–5]. KDIGO guidelines for kidney-transplant recipients state that treatment should be directed at the underlying cause. In contrast, regular testing for anemia is not recommended by the above-mentioned guidelines, and treatment of posttransplant anemia should be managed according to the guidelines for chronic kidney disease (CKD) in the predialysis period, with no specific recommendation for treatment of this specific population [2].

Additionally, the impact of the variability of hemoglobin over time in transplant recipients as compared with dialyzed patients has been considered in only a few studies. Some relationships between rejection episodes, immunosuppressant use, and increased anemia prevalence [9, 10], as well as between anemia of renal origin and mortality [11–13], have been shown.

Renal anemia after transplantation and its association with transplant outcomes have not been sufficiently explored; moreover, longitudinal studies on the association between anemia and mortality are rather rare, and PTA is still an underestimated problem [7, 13–15]. Therefore, the aim of this study was to explore whether anemia shortly after kidney transplantation predicts mortality at up to 10 years' follow-up.

## 2. Materials and Methods

### 2.1. Sample and Procedure

A total of 362 consecutive patients who underwent KT between January 2001 and January 2011 at the Transplant Centre of Kosice in the eastern region of Slovakia were enrolled in the study. The presented findings are part of a bigger study focused on quality of life measured using several questionnaires; the collection of medical data for this study took place during the collection of the questionnaires. The baseline examination of the participants took place between the 3rd and 12th month after successful KT during regular outpatient clinical visits in our centre. The inclusion criterion was graft survival at 3 months after KT, because the first 3 months after KT are usually considered as the most problematic period associated with dramatic changes and increased morbidity and even mortality [16]. All recipients were previously included on the waiting list for a kidney transplant. Therefore, they were tested for all serious comorbidities, such as cancer, which is always an exclusion criterion for transplantation; thus, no transplanted recipients had a cancer diagnosis at baseline. Additionally, the degree of renal anemia during a period shorter than 3 months after successful transplantation depends on the pre- and peritransplantation period [2]. In the case of any severe medical problem (infection, rejection, surgery, etc.) data collection was postponed by one month after overall clinical stabilization.

**Figure 1:**
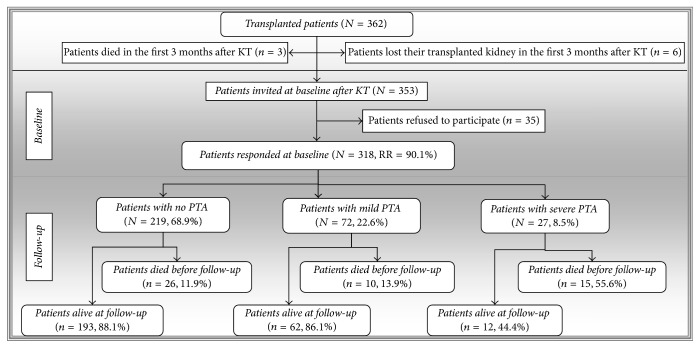
Flow-chart diagram of the participants. *N*/*n*: number; RR: response rate; KT: kidney transplantation; PTA: posttransplant anemia.

Nine patients dropped out prior to reaching 3 months after transplantation: 3 (0.8%) died and 6 (1.7%) lost their transplanted kidney. In total 353 kidney-transplant recipients after successful transplant surgery were invited to participate. Out of these, 35 (9.9%) refused to participate, resulting in a total of 318 patients (an effective response rate of 90.1%) at the start of the study.  Figure 1 presents more detailed information about the sample (Figure 1). Only patients who signed an informed consent form prior to the study were included. The Institutional Ethics Committee of the University Hospital in Kosice approved the study. All data and information used from the documentation, including demographic and clinical ones, were used in accordance with the ethical standards as laid down in the 1964 Declaration of Helsinki and its later amendments or comparable ethical standards.

### 2.2. Measures

#### 2.2.1. Sociodemographic Data

Sociodemographic data included age and gender. Age was treated as a continuous variable. Male gender was the reference category.

#### 2.2.2. Clinical Data

Clinical data were retrieved from medical files. These included serum hemoglobin, creatinine (laboratory methods by Scheffe), primary kidney diagnosis, previous duration of dialysis (in years), source of transplanted kidney, comorbidities, current and antirejection immunosuppressive treatment, acute rejection episodes, chronic renal allograft dysfunction, uroinfection (which included pyelonephritis and diagnosis of graft loss), and mortality. The estimated glomerular filtration rate (eGFR) was calculated using the CKD-EPI formula in milliliters per minute and 1.73 m^2^ [17]. CKD stages were determined as recommended by the “Kidney Disease Initiative for Global Outcomes” (KDIGO) guideline. This proposes a classification of chronic kidney disease [2, 6]; the classification reflects the impact of CKD (stages) for risk evaluation, diagnosis, patient management, and treatment options. In order to explore the effect of the CKD stages on anemia, we stratified the sample into two groups, as groups consisting of the separate CKD stages were too small for stratification: CKD stages 1-2 versus CKD stages 3–5, according to the known impact of deceased kidney function on increasing anemia of renal origin from CKD stage 3 [2, 6]. Acute rejection episodes and chronic renal allograft dysfunction were diagnosed from a biopsy according to the Banff 2009 update of diagnostic categories for renal allograft biopsies [18]. Depending on the hemoglobin value, PTA was divided into 3 categories: (1) severe PTA (Hb < 10 g/l), (2) mild PTA (10 ≤ Hb < 12 g/l), and (3) no PTA (Hb ≥ 12 g/dl) according to the “European Renal Best Practice” (ERBP) Guidelines [3, 4]. Severe cardiac failure was classified by the New York Heart Association (NYHA) Functional Classification as Classes III and IV [19].

#### 2.2.3. Mortality Data

Mortality data were obtained from our database of medical reports and completed with data from the “Health Care Surveillance Authority of the Slovak Republic” up to 10 years after KT.

### 2.3. Statistical Analyses

Frequencies, means, and standard deviations were calculated for the sample description. The Mann–Whitney *U* test and *χ*^2^ test were used to identify the association between mortality and the following baseline variables: age, gender, duration on dialysis before KT (in years), eGFR and CKD stages, PTA (severe, mild, and no anemia, which was the reference category), uroinfection (pyelonephritis included), number of acute rejection episodes, chronic renal allograft dysfunction, source of transplanted kidney, cardiovascular disease (coronary artery disease, cardiac failure, myocardial infarction), hypertension, and categories of diabetes mellitus (no diabetes mellitus, already existing diabetes mellitus, and new-onset diabetes mellitus after transplantation). Stratification by CKD was performed with regard to the known impact of decreased kidney function on anemia of renal origin [6] in order to study the potentially different associations between PTA and other incorporated variables independently of kidney function. These variables were included in the analysis due to the past research evidence outlined in the Introduction [2, 5]. Kaplan–Meier plots and log-rank test were used to display the differences between mortality risks by PTA categories separately for CKD stages 1-2 compared to CKD stages 3–5. Cox regression was performed in order to identify the predictors of mortality (censored for graft loss). The independent variables in both stratified Cox regression models were all variables with a level of significance set at *p* < 0.1 in the Mann–Whitney *U* test and the *χ*^2^ test, as appropriate. The Statistical Package for the Social Science (IBM SPSS Inc., Chicago, IL, USA) version 24 was used for statistical analyses.

## 3. Results

No significant differences were found at baseline between participants and nonparticipants regarding age, gender, graft loss, and mortality. The observation period of follow-up was from 1 to 10 years (mean 5.6 ± 2.7); the mean period for severe PTA was 4.3 ± 2.6 years, for mild PTA 5.3 ± 2.6 years, and for the category without PTA 5.9 ± 2.7 years. The prevalence of renal anemia therapy was 14% with Erythropoiesis-Stimulating Agents (ESA), 48% iron supplementation, 33% folic acid, 14% ascorbic acid, 10% pyridoxine, and 6% cobalamin.  Table 1 displays detailed information about the characteristics of the sample (*N* = 318) (Table 1).

**Figure 2:**
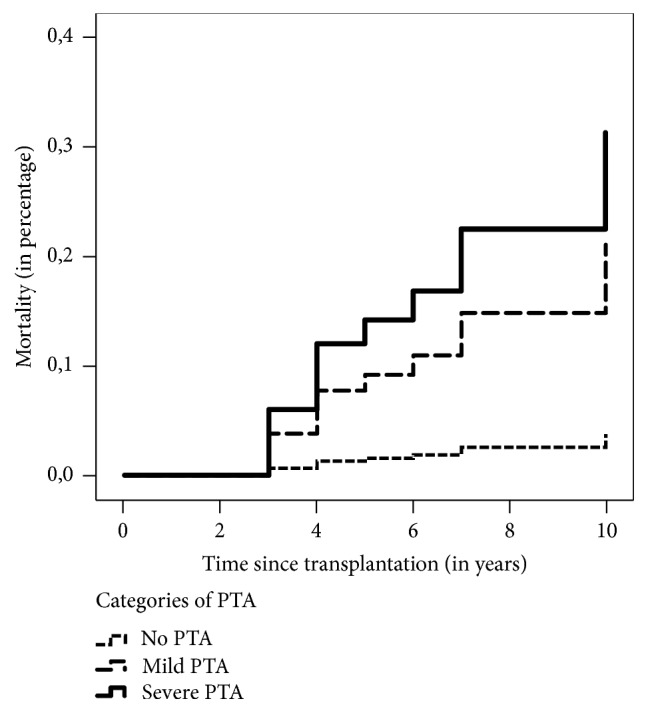
Differences in mortality between severe PTA, mild PTA, and no PTA over 10 years in CKD stages 1-2. Kaplan–Meier plots showing higher mortality during 10 years after transplantation in patients with CKD stages 1-2 with mild or severe anemia compared with patients without PTA. Log-rank test: *χ*^2^ = 39.62 and *p* value of the model < 0.001.

**Figure 3:**
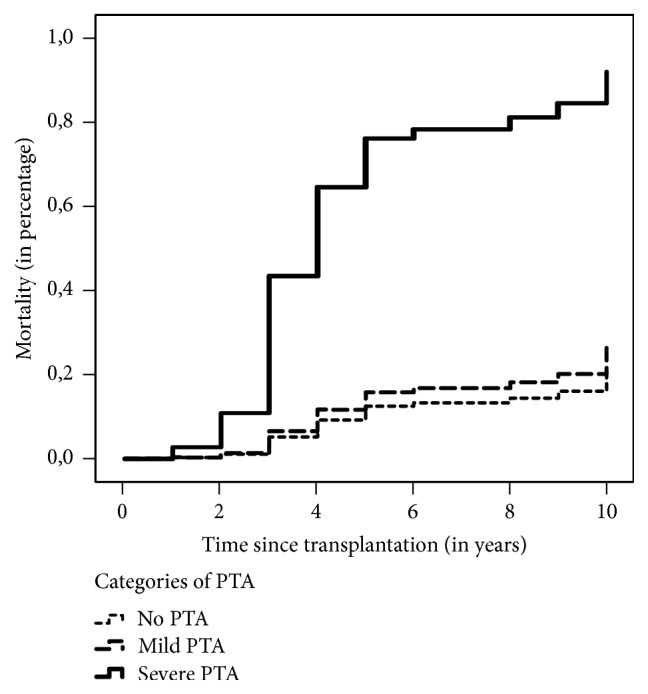
Differences in mortality between severe PTA, mild PTA, and no PTA over 10 years in CKD stages 3–5. Kaplan–Meier plots showing higher mortality during 10 years after transplantation in patients with CKD stages 3–5 with severe PTA compared with patients without PTA. Log-rank test: *χ*^2^ = 32.09 and *p* value of the model < 0.001.

The Mann–Whitney *U* test found that those who died were older (*p* < 0.001) and had a lower eGFR (*p* < 0.001) in the first year after KT. *χ*^2^ test indicated that they were also more likely to be of female gender (*p* < 0.1), to have a higher degree of PTA (*p* < 0.001), to have a more advanced stage of CKD (*p* < 0.001), and to show the presence of severe cardiac failure (*p* < 0.1) in the first year after KT (Table 1). The risk of death in patients with mild and severe PTA in CKD stages 1-2 compared with no PTA starts to rise at 3 years after KT; the hazard ratio for mild and severe PTA compared with no PTA increased after this period independently of kidney function. However, the risk of death in patients with severe PTA in CKD stages 3–5 starts to rise already at 2 years after KT. Figures 2 and 3 display the differences in mortality between those with severe PTA, those with mild PTA, and those with no PTA. (Figures 2 and 3).

### 3.1. Model 1: Cox Regression Model for Mortality in CKD Stages 1-2

Age (HR 1.1, *p* ≤ 0.001), female gender (HR 0.1, *p* ≤ 0.05), mild PTA (HR 6.2, *p* ≤ 0.05), and severe PTA (HR 9.8, *p* ≤ 0.001) contributed significantly to Cox regression model 1 for mortality in CKD stages 1-2. The risk of death increased by 10% for each year of age, while, on the other hand, the risk of death decreased by 90% among females. In addition, the presence of mild PTA increased the risk 6-fold and severe PTA 10-fold (Table 2).

### 3.2. Model 2: Cox Regression Model for Mortality in CKD Stages 3–5

Age (HR 1.1, *p* ≤ 0.01) and severe PTA (HR 10.8, *p* ≤ 0.001) contributed significantly to Cox regression model 2 for mortality in CKD stages 3–5. The risk of death increased by 10% for each year of age, and the presence of severe PTA increased the risk 10-fold (Table 2).

## 4. Discussion

We explored the independent effect of anemia on mortality in the early period after kidney transplantation. Mild and severe PTA in the first year after transplantation increased the higher risk of mortality independently of kidney function at up to 10 years' follow-up. Mild PTA predicted a 6-fold higher risk of mortality and severe PTA a 10-fold higher risk of mortality compared with no PTA in CKD stages 1-2. However, patients with more advanced stages of CKD showed no association of mild PTA with mortality, probably as this only reflects their worse kidney function; however, severe PTA predicted a 10-fold higher risk of mortality. The other factor associated with increased risk of mortality was advanced age and that with decreased mortality was female gender, which is in line with the other studies [20, 21].

NKF/KDOQI and KDIGO guidelines for diagnosis and treatment of renal anemia recommend a global assessment of the patient, which should consist of an inventory of complications of the dialyzed, perioperative, and posttransplantation period, including inflammatory diseases, rejections, comorbidities, ACEi/ARB, and immunosuppressant treatment [2, 3, 6]. In our study uroinfection (including pyelonephritis), rejection episodes, chronic renal allograft dysfunction, cardiovascular disease, already existing diabetes mellitus, new-onset diabetes mellitus after transplantation, and the total number of other comorbidities were not associated with mortality in patients after KT.

In line with our results, Amaral et al. showed that patients with mild and severe anemia of renal origin independently of CKD had an increased risk for mortality [22]. A few other studies have also shown that a low level of Hb is strongly associated with mortality [7, 12, 13]. In our sample, 31.1% of respondents had various grades of anemia, fitting in the range from 20 to 57%, as was found earlier for Central Europe [23].

The study of Lawler et al. (2010), with CKD in the predialyzed period, showed that anemia in this group was underestimated, with an absence of relevant blood tests and a lack of treatment [24]. Iseki and Kohagura showed that renal anemia is a marker of kidney failure and is associated with a higher incidence of stroke and heart failure and relevant lower quality of life and survival [25]. Regarding the above-mentioned outcomes, Amaral et al. suggested that the hazard ratio of mortality is increased proportionally according to the severity of the anemia. They discovered that a serum hemoglobin concentration of 11.0 g/dl and higher showed a 60–70% reduction in the risk of mortality [22].

The prevalence of renal anemia treatment after KT is, according to the NKF/KDOQI guidelines, relevant to CKD in the predialyzed period [5]. Surprisingly, Molnar et al. found in ten renal transplant units across Europe that the prevalence and management practices related to renal anemia after transplantation were quite variable and overall have remained largely unchanged over the last 5 years [11]. In our sample, more than two-thirds of the patients were treated by a combination of two drugs. Similar to our results, Spiegel and Chertow (2009) showed the benefit of renal anemia treatment by ESA and iron therapy [8].

The most recent studies regarding renal anemia therapy have shown that there is a narrow boundary between safe treatment and therapy causing increased morbidity and mortality risk [26]. Reports on the relative adequate serum concentration of hemoglobin and iron and other essential components have shown a higher risk of stroke, thrombosis, and progression of cancer [26, 27]. These conclusions bring up new questions about dosing algorithms aimed at achieving and maintaining optimal target hemoglobin levels without endangering the patient.

### 4.1. Strengths and Limitations

The main strength of this study is the prospective follow-up for 10 years, which enabled us to explore anemia and other factors as predictors of mortality in kidney-transplant recipients. Moreover, all consecutive patients originating from one major transplant centre in Slovakia over a number of years were asked to participate in the study to prevent selection bias. Additional strengths of this study are the exclusion of problems in the first 3 months, the long follow-up, and the exclusion of preexisting cardiovascular disease and rejection episodes. Moreover, this study compares the PTA impact on mortality separately for well-functioning graft versus advanced stages of CKD and addresses the underreporting and undertreating of renal anemia in patients with a well-functioning graft (CKD1-2). The findings demonstrated the ability of using PTA in patients with a well-functioning graft for predicting mortality.

In contrast, stratification of the sample into only two groups according to CKD stages is the main limitation of this study. We were unable to compare each CKD stage regarding the mortality prediction by anemia separately due to the low number of participants in CKD stages four and five. Additionally, the level of PTA was based on the Hb-value at baseline. The next limitation of the study might be the lack of certain other biomarkers (serum concentration of ESA, iron, ferritin, transferrin, vitamins, etc.) and additional information (inflammatory markers, catabolism). These factors, therapy, and inflammatory markers are associated with a decrease in the hemoglobin value; in addition, they play an important role in exploring the causes of PTA and in its incidence. Therefore, these factors have to be considered in future research. The variable observation period between minimum and maximum (1 and 10 years) is also a limitation. Testing for anemia in this study was not conducted immediately after transplantation to prevent false findings due to perioperative complications. Therefore, patients who died or lost their transplanted kidney within the first 3 months after KT were not included in the study. It could be of interest to control for a potential effect of pretransplantation anemia and potential progression of PTA, as they may predict mortality and graft loss, as well.

### 4.2. Recommendations

Our findings imply that mild and severe anemia in CKD stages 1-2 may be an independent element of the pathway to survival in kidney-transplant recipients. In line with our results, we suggest treating mild and severe anemia in patients after the third month following successful transplantation to increase their probability for survival. Further studies should also be carried out to shed more light on this important pathway. It would be worthwhile to plan a similar study based on the individual CKD stages in the future. According to these results, a randomized controlled trial in ESA treatment of posttransplant anemia with a target Hb-value above 10.0 g/dl would be appropriate. We could then verify whether treatment of anemia after KT decreases mortality in kidney-transplant recipients and thus fills a gap in the guidelines for ESA in posttransplant anemia regarding the Hb-value. Furthermore, the pathways between other medical determinants associated with anemia and mortality should be studied as well.

## 5. Conclusion

Posttransplant anemia in an early period after transplantation increased the risk of mortality independently of kidney function at up to 10 years' follow-up in CKD stages 1-2. Mild PTA is associated with a 6-fold higher risk of mortality and severe PTA with a 10-fold higher risk of mortality compared with no PTA in CKD stages 1-2. Thus, patients with a well-functioning transplanted kidney but with posttransplant anemia might benefit from clinical evaluation as well as treatment (e.g., Erythropoiesis-Stimulating Agents, iron therapy) to reduce their higher risk of mortality. However, patients with more advanced stages of CKD showed no association of mild PTA with mortality, probably as this may only reflect their worse kidney function; however, severe PTA predicted a 10-fold higher risk of mortality.

## Figures and Tables

**Table 1:** Characteristics of the sample (*N* = 318).

Characteristics of the sample at baseline	Died after transplant (*n* = 51)	Survived after transplant (*n* = 267)	*p* value
	*n* (%) or mean ± SD	*n* (%) or mean ± SD	
Age	48.4 ± 6.8	46.9 ± 7.4	0.001

Gender			
Male	24 (47.1%)	159 (59.6%)	0.05
Female	27 (52.9%)	108 (40.4%)	

Duration on dialysis before KT (in years)	3.7 ± 2.1	3.5 ± 2.9	n.s.

Primary diagnosis of kidney failure			
Glomerulonephritis	19 (37.3%)	96 (36.0%)	n.s.
Tubulointerstitial nephritis	12 (23.5%)	66 (24.7%)	
Vascular disease	3 (5.9%)	28 (10.5%)	
Polycystic kidneys adult type	2 (3.9%)	19 (7.1%)	
Diabetic nephropathy	8 (15.7%)	13 (4.9%)	
Others or unknown	7 (13.7%)	45 (16.8%)	

Source of transplanted kidney			
Deceased donor	47 (92.2%)	256 (95.9%)	n.s.
Living donor	4 (7.8%)	11 (4.1%)	

Function immediately after KT			
Immediate function	27 (52.9%)	150 (56.2%)	n.s.
Delayed function	24 (47.1%)	117 (43.8%)	

Estimated glomerular filtration rate (ml/min/1.73 m^2^)	61.2 ± 19.8	63.8 ± 20.1	0.001

CKD stage			
1	5 (9.8%)	18 (6.7%)	0.07
2	20 (39.2%)	115 (43.2%)	
3a + 3b	21(41.2%)	101 (37.8%)	
4	3 (5.9%)	11 (4.1%)	
5	2 (3.9%)	22 (8.2%)	

Hemoglobin value (g/dl)	11.9 ± 1.9	12.7 ± 2.1	0.001

Posttransplant anemia			
Severe (Hb < 10.0 g/dl)	8 (15.8%)	19 (7.1%)	0.001
Mild (10.0 ≤ Hb < 12.0 g/dl)	17 (33.3%)	55 (20.6%)	
No anemia (Hb 12.0 g/dl)	26 (50.9%)	193 (72.3%)	

Therapy for anemia			
ESA	3 (5.9%)	11 (4.1%)	n.s.
Iron	8 (15.7%)	39 (14.6%)	
Folic acid	14 (27.4%)	19 (7.1%)	
Cobalamin	2 (3.9%)	4 (1.5%)	
Pyridoxine	1(2.0%)	9 (3.4%)	
Ascorbic acid	3 (5.9%)	11 (4.1%)	

Acute rejection episodes	17 (33.3%)	78 (29.2%)	n.s.

Type of rejection treatment			
Steroids	9 (17.6%)	63 (23.6%)	n.s.
Antithymocyte globulin	2 (3.9%)	8 (3.0%)	
Plasmapheresis	1(2.0%)	5 (1.9%)	
Plasmapheresis + i.v. immunoglobuline	1(2.0%)	6 (2.2%)	

Chronic renal allograft dysfunction	8 (15.7%)	34 (12.7%)	n.s.

Uroinfection (including pyelonephritis of graft)	14 (27.4%)	71 (26.6%)	n.s.

Immunosuppression treatment			
CsA + P	7 (13.7%)	29 (10.9%)	n.s.
CsA + AZA/CsA + AZA + P	8 (15.7%)	16 (6.0%)	
CsA + MMF/CsA + MMF + P	23 (45.1%)	131 (49.0%)	
Tac + MMF/Tac + MMF + P	11 (21.6%)	86 (32.2%)	
SIR + MMF + P/EVER + CsA + MMF	2 (3.9%)	5 (1.9%)	

Comorbidities			
Coronary artery disease	11 (21.6%)	67 (25.1%)	n.s.
Severe cardiac failure	15 (29.4%)	57 (21.3%)	0.09
Myocardial infarction	3 (5.9%)	14 (5.2%)	n.s.
Hypertension	37 (72.5%)	189 (70.8%)	n.s.
Diabetes mellitus identified before KT	8 (15.7%)	22 (8.2%)	n.s.
NODAT	3 (5.9%)	14 (5.2%)	n.s.
CKD-MBD	23 (45.1%)	140 (52.4%)	n.s.
Other comorbidities: ≥2	1(2.0%)	8 (3.0%)	n.s.

Level of significance *p* < 0.1; *N*/*n*: number, SD: standard deviation, AZA: azathioprine, CKD: chronic kidney disease, MBD: mineral bone disorder, NODAT: new-onset diabetes mellitus after transplantation, CsA: cyclosporine A, ESA: erythropoiesis-stimulating agents, EVER: everolimus, Hb: hemoglobin, KT: kidney transplantation, n.s.: not significant, MMF: mycophenolate mofetil/mycophenolate sodium, P: prednisone, SIR: sirolimus, and Tac: tacrolimus.

**Table 2:** Final models of Cox regression [stratified due to 2 CKD groups (CKD stages 1-2 and 3–5)] containing predictors of mortality.

Models for mortality (*N* = 318)	HR	95% CI for HR	*p* value
*Model 1 in CKD stages 1-2* (*n* = 158)			

Age	1.12	1.05; 1.20	0.000

Gender			
Male	Reference		
Female	0.09	0.09; 0.82	0.033

Severe cardiac failure			
No	Reference		
Yes	1.82	0.60; 4.23	0.316

PTA			
No	Reference		
Mild	6.16	1.12; 34.33	0.038
Severe	9.79	2.57; 37.26	0.001

*Model 2 in CKD stages 3–5* (*n* = 160)			

Age	1.06	1.02; 1.09	0.003

Gender			
Male	Reference		
Female	0.59	0.29; 1.20	0.593

Severe cardiac failure			
No	Reference		
Yes	1.92	0.82; 5.56	0.835

PTA			
No	Reference		
Mild	1.29	0.56; 2.98	0.554
Severe	10.78	4.15; 28.08	0.000

PTA: posttransplant anemia, CI: Confidence Interval, HR: hazard ratio.
